# Cytotoxic Effects of a Triorganotin Derivative on HTLV-1-Infected Cells at Different Immortalization/Transformation Stages In Vitro

**DOI:** 10.3390/molecules31020349

**Published:** 2026-01-19

**Authors:** Valeria Stefanizzi, Antonella Minutolo, Evariste Molimbou, Emanuela Balestrieri, Martina Giudice, Franca M. Cordero, Claudia Mosca, Antonio Mastino, Beatrice Macchi, Claudia Matteucci, Sandro Grelli, Francesca Marino-Merlo

**Affiliations:** 1 Department of Chemical, Biological, Pharmaceutical, and Environmental Sciences, University of Messina, I-98166 Messina, Italy; valeria.stefanizzi@unime.it (V.S.); claudia.mosca@unime.it (C.M.); francesca.marino@unime.it (F.M.-M.); 2 Department of Experimental Medicine, University of Rome “Tor Vergata”, I-00133 Rome, Italy; antonellaminutolo@gmail.com (A.M.); emolimbou@gmail.com (E.M.); balestrieri@med.uniroma2.it (E.B.); giudicemartina94@gmail.com (M.G.); matteucci@med.uniroma2.it (C.M.); grelli@med.uniroma2.it (S.G.); 3 Department of Chemistry “Ugo Schiff”, University of Florence, I-50019 Sesto Fiorentino, Italy; franca.cordero@unifi.it; 4 The Institute of Translational Pharmacology, Consiglio Nazionale delle Ricerche, I-00133 Rome, Italy; 5 Department of Chemical Science and Technology, University of Rome “Tor Vergata”, I-00133 Rome, Italy

**Keywords:** HTLV-1, adult T-cell Leukemia/Lymphoma, organotin compounds, cell death

## Abstract

Among the metal-derived complexes, recently, tin derivatives have been investigated as promising anti-cancer drug candidates. Our previous study showed that the tin-based compound Bu_3_SnOCOCF_3_ (TBT) exerts cytotoxic activity on solid tumor cell lines. In the present study, the effects of TBT were evaluated in vitro on HTLV-1-infected human lymphocytic cell lines at different stages of viral transformation, consisting of IL-2-dependent (PB2/IL-2) and IL-2-independent (PB2/NO-IL-2) cells, generated in our laboratory by HTLV-1 in vitro infection of lymphocytes from the same donor, and the C91/PL cell line established by co-cultivation with T cells from a patient with HTLV-1-positive leukemia. TBT induced a reliable and reproducible dose-dependent inhibition of metabolic activity and viability in the HTLV-1-infected cells. The effect was cell-type-dependent, with C91/PL cells being quite resistant. An investigation into the cytotoxic effects induced by TBT in HTLV-1-infected cells and data on caspase inhibitors/caspase activation indicated that apoptotic cell death was involved, but also that the possible involvement of other forms of cell death could not be excluded. Taken together, the results show for the first time that the tin-based compound, although not devoid of a certain cytotoxicity toward uninfected cells, can induce typical and potent effects on HTLV-1-infected cells.

## 1. Introduction

Within the class of drugs defined as cancer chemotherapeutics, metal-derived platinum complexes are highly utilized for the treatment of solid tumors. However, platinum-derived drugs, like cisplatin, carboplatin, and oxaliplatin, while effective for the treatment of various tumors, are also known to cause significant side effects and to encounter drug resistance [1]. Recently, organotin derivatives have gained importance in the development of metallopharmaceutics, owing to the flexibility of their molecular structure, allowing for combination with structures of different origins, and to their potential interaction with cell macromolecules such as DNA, inducing cell damage or loss of protein folding by interacting with free SH groups [2]. Therefore, organotin compounds have been investigated as promising anti-tumor drugs, due to their ability to contrast cancer cell growth/proliferation in vitro. The synthesis of organotin (IV) complexes linked to carbamate, carboxilated acid, or hydroxamic acid provided compounds endowed with anti-tumoral effects. The mechanisms involved in the anti-tumor effects of tin-based compounds have been the object of various studies. Recently, it was shown that a series of organotin (IV) compounds linked to different dithiocarbamate groups were lipophilic and induced cytotoxicity, apoptosis, and cell cycle arrest, mainly at the S-G2/M phase in a model of T acute lymphoblatic leukemia, Jurkat E6.1 [3]. Conversely, thriphenyltin (IV) carboxylate derivatives of non-steroidal anti-inflammatory drugs were cytotoxic in breast cancer cell lines in vitro, suppressing NO production through inhibition of the iNOS enzyme [4], while other carboxylated derivatives induced cell cycle arrest, inhibiting cell migration by affecting ROS production and mitochondrial membrane potential [5]. In addition, organotin benzohydroxamate compounds (OTBH) with different electronegativity of fluorine and chlorine atoms were found to target microtubules in adherent cells of different origins, and this was hypothesized to be the triggering signal capable of inducing cell death by apoptosis [6]. This evidence highlights how organotin-induced death might depend on the organotin structure, on the molecular target, and on a variegate involvement of cellular death pathways.

T Human T-Lymphotropic virus type 1 (HTLV-1) is prevalent in endemic areas encompassing mainly Japan, South and Central America, Australia, and Sub-Saharan Africa, accounting for an estimated 5–10 million infected individuals worldwide [7,8,9,10,11,12]. Except for Romania, which was recognized as an endemic area for HTLV-1 in the early 1990s [13], Europe is not considered an endemic area, but owing to immigrant flux from endemic areas, HTLV-1 is tracked in England, France, and Spain [14,15]. In other European countries, HTLV-1 is not tracked. For example, however, simply based on foreigners officially registered as resident in Italy and from data on HTLV-1 prevalence among the general population in the corresponding countries, it was recently calculated that there are approximately 26,000 neglected HTLV-1-positive foreigners living in the country [16]. HTLV-1 is the causative agent of two distinct pathologies, adult T-cell leukemia/lymphoma (ATL) and tropical spastic paraparesis/HTLV-1-associated myelopathy (TSP/HAM). The virus preferentially infects CD4+ T cells, but it also hijacks CD8+ CTLs, NK cells, and monocytes/macrophages, hindering the immune response toward the virus and favoring its spread to CD4+ T cells [17,18,19,20,21,22]. Infection is characterized by a life-long latency driven by alternate blips of cell replication and cell death [23]. The main, natural route of virus transmission is mother to child transmission, mainly through breastfeeding, but it can also be transmitted through sexual contact and blood-to-blood contact [24].

Currently, there are no effective therapies or vaccines to prevent or definitely counteract infection or diseases caused by HTLV-1. Nevertheless, a triple-agent regimen, consisting of arsenic trioxide as chemotherapeutic agent and interferon-alpha and zidovudine as immunotherapeutic and antiviral agents, resulted in a high rate of response in newly diagnosed chronic ATL patients [25]. The mechanisms involved in the therapeutic effectiveness of this triple combination, however, were not exactly clear. The results of the above-mentioned and other clinical trials, together with those recently obtained in a number of in vitro studies on the complex oncogenesis triggered by HTLV-1, suggest that novel, multi-target therapeutic strategies focused on the selective targeting of both viral factors required for viral replication and transformation, and host factors required for cell proliferation/death and immunity, must be explored in the near future to counteract diseases caused by HTLV-1, especially when administered early in the progression of the disease [26,27,28,29,30,31].

Our previous data have shown that tributyltin (IV) inhibited cell growth through multiple mechanisms characterized by a co-existence of apoptosis and necrosis in HSSC head and neck squamous carcinoma cell lines [32]. In this study, we explored the effect of an ad hoc synthesized organotin derivative chosen as a prototype tin-based compound (Bu_3_SnOCOCF_3_, hereinafter simply termed as TBT) on HTLV-1-infected immortalized/transformed cell lines, as a model of virus-driven oncogenesis undergoing prolonged virus-mediated changes in the target cells after infection, ultimately leading to transformation. This was performed by attempting to mimic in vitro the natural, malignant progression of lymphoid cells infection by HTLV-1, starting with an early stage of factor-dependent growth, continuing with a factor-independent stage of immortalization, and ending with a transformation stage. Different parameters were assessed to demonstrate whether the prototype organotin compound was able to differently affect infected cells’ metabolic rate, growth, viability, and death at different stages of infection/transformation.

## 2. Results

### 2.1. Flow Cytometry Phenotypic Characterization of HTLV-1-Infected Cell Lines at Different Stages of Immortalization/Transformation

**Figure 1:**
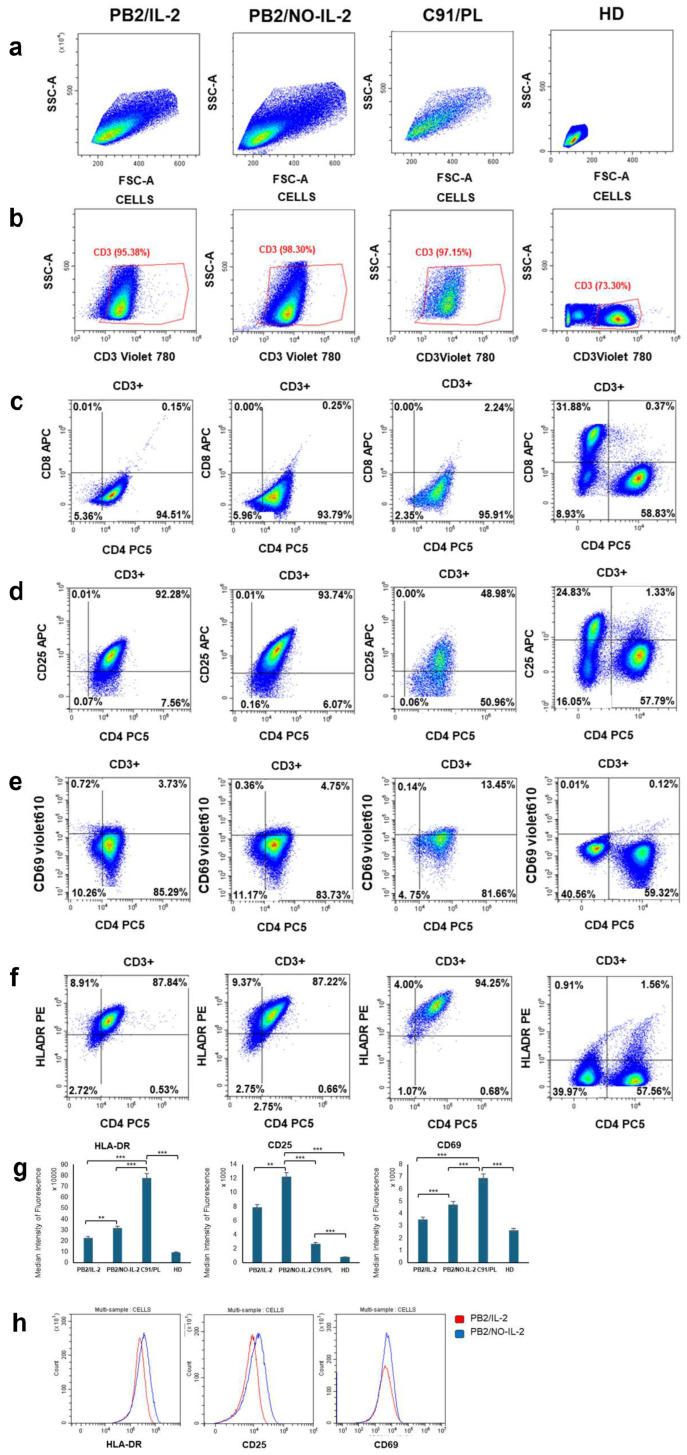
Flow cytometry analysis of T-cell subsets and activation markers in HTLV-1-infected and uninfected cells. Representative dot plots showing gating strategy and expression of activation markers in CD3^+^ T lymphocytes from HTLV-1-infected and -immortalized PB2/IL-2 and PB2/NO-IL2 cells, HTLV-1-transformed C91PL cells, and PBMCs from a healthy donor (HD) are reported. Row (**a**) shows the initial gating of total cells based on FSC-A and SSC-A parameters. Row (**b**) illustrates CD3^+^ T-cell selection. Subsequent dot plot panels display CD4^+^ and CD8^+^ T-cell distribution (**c**) and the expression of activation markers CD25 (**d**), CD69 (**e**), and HLA-DR (**f**) within the CD3^+^ gate. Bar graphs in row (**g**) show the median fluorescence intensity of HLA-DR, CD25, and CD69 in HTLV-1-infected cells and in cells from a HD. Data represent the mean values ± S.D. of at least three independent experiments. Statistical significance was calculated using Student’s *t*-test (** *p* < 0.05, *** *p* < 0.01). Representative histograms in row (**h**) graphically illustrate fluorescence intensity for HLA-DR, CD25, and CD69 in PB2/NO-IL-2 cells (blue peaks) compared with PB2/IL-2 cells (red peaks).

To mimic in vitro different stages of virus-induced immortalization/transformation, we utilized an IL-2-dependent cell line, designated as PB2/IL-2, and an IL-2-independent cell line, designated as PB2/NO-IL-2, generated in our laboratory. The PB2/IL-2 cell line was established by in vitro infection with HTLV-1 of peripheral blood lymphocytes from a single healthy donor and continuous cell culture in the presence of the IL-2 growth factor for 12 months before frozen stockage. PB2/NO-IL-2 was established, in turn, during the course of this study from freshly thawed PB2/IL-2 by continuos cell culture and progressive extremely low reduction in the concentration of IL-2 in the culture medium before successive stabilization in culture for a further 12 months. Before subjecting PB2/IL-2 and PB2/NO-IL-2 to TBT treatment, the cell lines underwent a basic phenotypic characterization via flow cytometry to verify possible differences in their T-cell activation status and absence of contaminating cell types. For comparison, the C91/PL-transformed cell line and uninfected peripheral blood mononuclear cells (PBMCs) from a healthy donor (HD) were utilized. Representative dot blots of T-cell subsets, reported in Figure 1b,c, clearly exclude the presence of contaminating cells in PB2/IL-2, PB2/NO-IL-2, or C91/PL cells, with practically all these cells being CD3 and CD4 positive, in contrast with HD PBMCs. Moreover, all the HTLV-1-infected cell lines expressed the CD25, CD69, and HLA-DR activation markers at higher levels with respect to CD4+ cells from a HD (Figure 1d–f). Notably, median fluorescence analysis revealed a common trend in the cells toward higher expression levels for all activation markers in the following order: HD PBMCs, PB2/IL-2, PB2/NO-IL-2, and C91/PL, except for CD25 in C91/PL cells (Figure 1g,h). 

### 2.2. Effects of TBT on the Metabolic Rate in HTLV-1-Infected Cells

**Figure 2:**
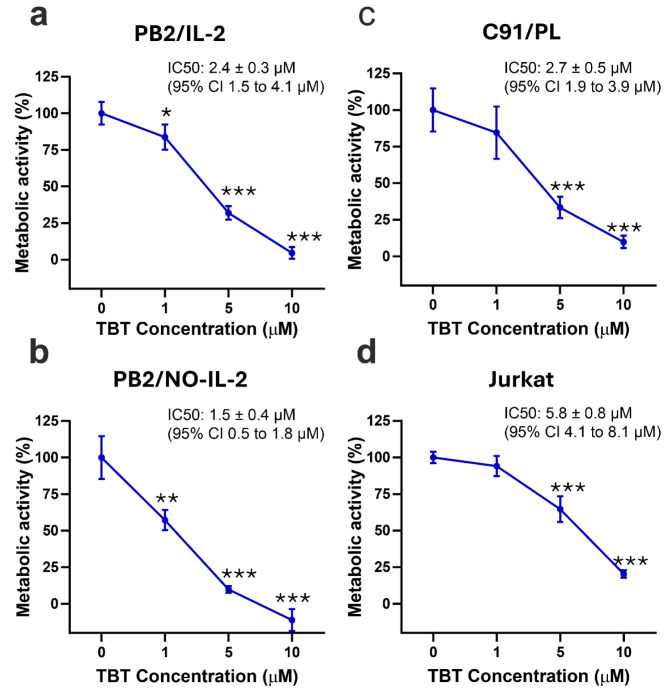
Inhibition of metabolic activity, detected by WST-1 assay, in PB2/IL-2 (**a**), PB2/NO-IL-2 (**b**), C91/PL (**c**), and Jurkat (**d**) cell lines after treatment for 24 h with control diluent (0), 1, 5, or 10 µM TBT. The results are expressed as mean values ± S.D. obtained from three independent experiments, each in triplicate. IC_50_ values were calculated via nonlinear regression and are reported in the figure together with their 95% confidence intervals (CI). Asterisks indicate statistically significant comparisons versus diluent-treated control (* *p* ≤ 0.05, ** *p* ≤ 0.01, *** *p* ≤ 0.001). IC50 value is the concentration of the compound that decreases metabolic activity by 50% compared to the control. Measurements were performed using a plate reader at 450 nm wavelength.

To verify the effects induced by TBT on the metabolic activity of HTLV-1-infected cells, the WST-1 assay for metabolic activity detection was utilized. As HTLV-1 infected lymphocytic cell targets at different stages of immortalization, PB2/IL-2 and PB2/NO-IL-2 cell lines were utilized. As HTLV-1 infected transformed cell targets, the C91/PL cell line was utilized. The Jurkat cell line was utilized as a reference, uninfected CD4+ lymphoid T-cell line, while peripheral blood mononuclear cells (PBMCs) from a healthy donor were utilized as a further control. The HTLV-1-infected cells were seeded and treated with concentrations of the TBT molecule ranging from 1 µM to 10 µM for 24 h before being subjected to the WST-1 assay. The inhibition of the metabolic activity was then evaluated arbitrarily assuming that in the control samples there was 100% metabolic activity. The results showed that TBT efficiently inhibited the metabolic activity of PB2/IL-2 cells with an IC50 of 2.4 ± 0.3 µM (Figure 2a). In PB2/NO-IL-2 cells a remarkable dose-dependent inhibition of the metabolic activity at all concentrations of TBT was detected, with an IC50 of 1.5 ± 0.4 µM (Figure 2b).

Regarding the HTLV-1-chronically infected C91/PL cell line, the results showed a sensitivity of these cells to the inhibition of metabolic activity by TBT, quite similar to what was observed for PB2/IL-2 cells, but considerably lower than what was observed for PB2/NO-IL-2, with an IC50 of 2.7 ± 0.5 µM (Figure 2c). Taken together, these data show that, considering the metabolic activity an indirect estimation of both viable and proliferating cells, the effect of TBT on PB2/IL-2 and C91/PL cells was equally properly cytostatic/cytotoxic, while it seemed to be more potent in PB2/NO-IL-2 cells. When the metabolic activity following TBT treatment was measured in the uninfected Jurkat T-cell line, the results showed that TBT inhibited the metabolic activity at higher concentrations with respect to what was observed in HTLV-1-infected cells, with an IC50 of 5.8 ± 0.8 µM (Figure 2d). An even higher IC50 of 6.39 ± 0.9 µM was obtained when PBMCs from an uninfected healthy donor were subjected to the same treatment with TBT and the same protocol of cell lines to evaluate the metabolic activity.

### 2.3. Effects of TBT on Viability/Death in HTLV-1-Infected Cells

**Figure 3:**
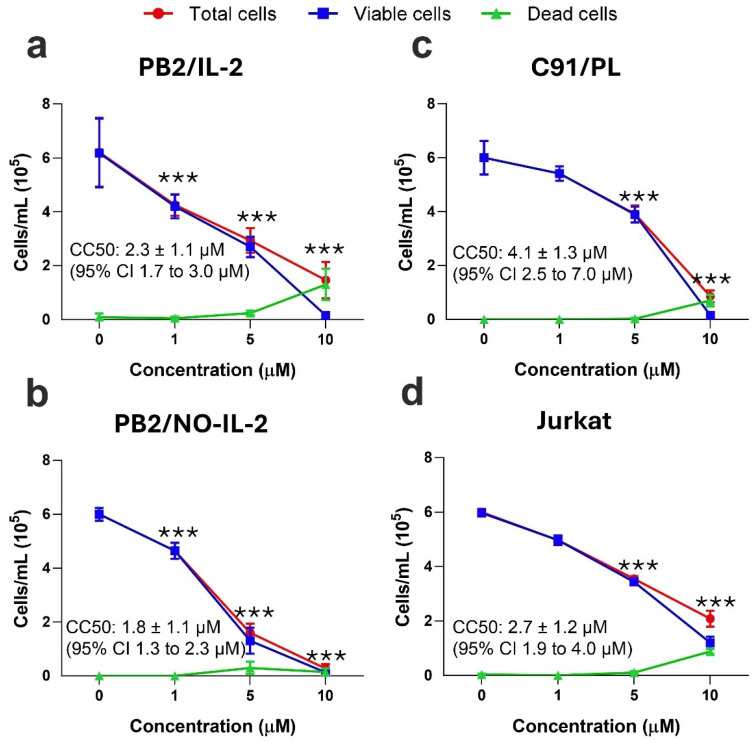
Cell death/viability, detected by the Trypan Blue exclusion test, in PB2/IL-2 (**a**), PB2/NO-IL-2 (**b**), C91/PL (**c**), and Jurkat (**d**) cell lines after treatment for 24 h with control diluent (0), 1, 5, or 10 µM TBT. The results are expressed as mean values ± S.D. obtained from three independent experiments, each in triplicate. IC_50_ values were calculated via nonlinear regression and are reported in the figure together with their 95% confidence intervals (CI). Asterisks indicate statistically significant comparisons of viable cell values in treated samples versus those of diluent-treated control (*** *p ≤* 0.001). CC50 value is the concentration of the compound that decreases cell viability by 50% compared to that of the control.

The same cellular targets used for evaluating the effects of TBT on the metabolic rate were also utilized to evaluate viability/death in HTLV-1-infected cells following TBT treatment. The classic Trypan Blue exclusion assay was employed, and the total number of still-intact viable/dead cells was evaluated following 24 h of treatment with different amounts of TBT. In PB2/IL-2, a progressive decrease in cell viability occurred at 1 µM and 5 µM of TBT, while at 10 µM there was an almost total disappearance of viable cells, with a CC50 of 2.3 ± 1.1 µM (Figure 3a). The results of the same assay carried out in the PB2/NO-IL-2 cell line are shown in Figure 3b. In this case, the response of PB2/NO-IL-2 cells to TBT treatment was slightly higher than that observed for PB2/IL-2 cells, with a CC50 value of 1.8 ± 1.1 µM. Note that in both cases dead cells were detectable at 24 h post treatment only in samples treated with 5 and 10 µM TBT and that, except for PB2/IL-2 at 10 µM, the number of Trypan Blue-positive dead cells in TBT-treated experimental samples was quite low and did not mirror the number of live cells. This suggests that at 24 h post treatment, many treated cells already underwent a more-or-less early form of lytic cell death, such as primary or secondary necrosis, and no longer consisted of intact, microscopy-detectable cells. Nevertheless, results could also be related to an early and strong inhibition of cell proliferation exhibited by TBT, as shown by experiments on inhibition of the metabolic activity (Figure 2). The HTLV-1-transformed C91/PL cell line showed a certain resistance to the toxic effect of TBT, as evaluated by the Trypan Blue assay, with a CC50 value calculated for the death/viability assay of 4.1 ± 1.3 µM (Figure 3c). When the dead/viable cells following treatment were evaluated in the uninfected lymphoid Jurkat T-cell line, the results showed that TBT inhibited viability at concentrations lower than those observed in C91/PL cells, but higher than those observed in PB2/IL-2 or PB2/NO-IL-2 cells, with a CC50 of 2.7 ± 1.2 µM (Figure 3d).

### 2.4. Flow Cytometry Analysis of Hypodiploidy in Samples from HTLV-1-Infected PB2/IL-2, PB2/NO-IL-2 After TBT Treatment

**Figure 4:**
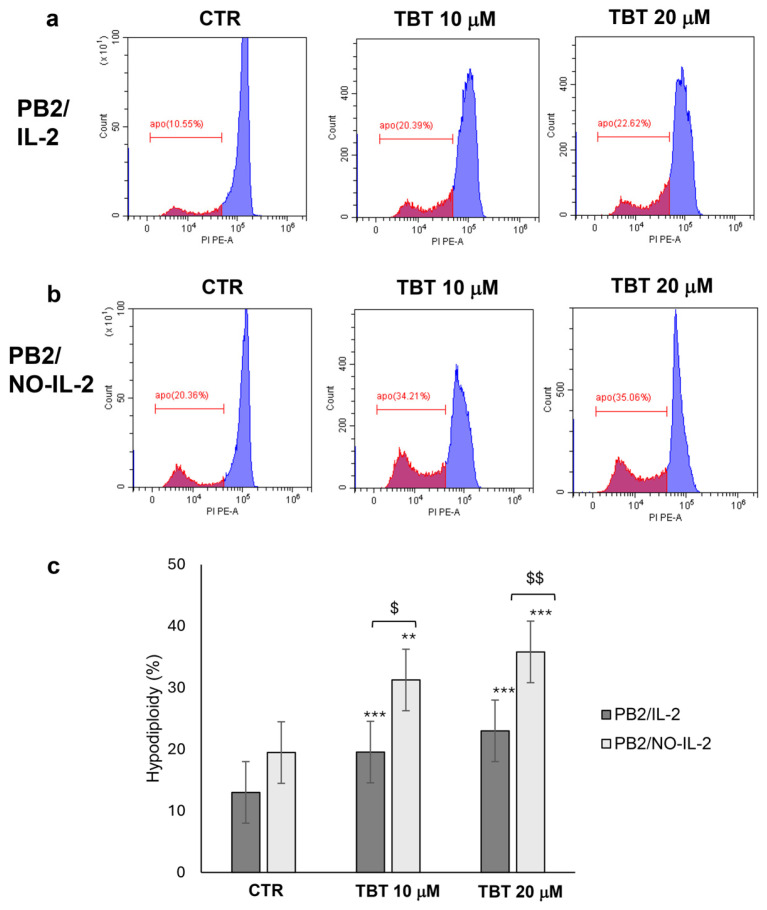
Hypodiploidy detected by flow cytometry analysis after propidium iodide staining in samples from PB2/IL-2 and PB2/NO-IL-2 cells after treatment for 13 h with control diluent (CTR), 10 µM, and 20 µM TBT. Representative histograms showing % hypodiploidy (red area) detected in control and treated samples from PB2/IL-2 (**a**) or PB2/NO-IL-2 (**b**) are shown. Results from three biological replicates, expressed as mean ± S.D. % hypodiploidy are reported in the bar graph (**c**). Asterisks and dollars indicate statistically significant comparisons in treated samples versus corresponding controls (** *p ≤* 0.05, *** *p ≤* 0.001) and between groups ($ *p ≤* 0.05, $$ *p ≤* 0.001), respectively.

With the aim of obtaining information on cell death induced by TBT in HTLV-1-infected cells, samples from PB/IL-2 and PB2/NO-IL-2 cell lines treated or not treated with the organotin compound were subjected to flow cytometry analysis following staining with the DNA-binding fluorescent dye propidium iodide (PI). This technique is a well-known traditional approach to detect apoptotic regulated cell death (RCD) by evaluation of nuclear hypodiploidy. Based on the results obtained with the Trypan Blue assay and preliminary experiments, to preclude as far as possible the presence of cells at a too advanced state of disruption but, at the same time, at a yet-undetectable state of cell death, this set of experiments was performed at 13 h post treatment. Representative flow cytometry histograms showing the effects of exposure to 10 µM and 20 µM of TBT compared with untreated DMSO controls are shown in Figure 4. The upper panels (Figure 4a) show PB2/IL-2 cells, whereas the lower panels (Figure 4b) correspond to PB2/NO-IL-2 cells. The red area indicates the hypodiploidy signals. The bar graph (Figure 3c) summarizes the numerical results, expressed as % hypodiploidy, and statistically significant differences in the groups from three independent experiments are also reported. TBT induced a dose-dependent increase in % hypodiploidy in both PB2/IL-2 and PB2/NO-IL-2 cells, with a difference that in any case was significant in respect to controls. Regarding intergroup differences, PB2/NO-IL-2 cells exhibited a higher sensitivity to TBT in comparison with PB2/IL-2, particularly at 20 µM. However, these cells showed also a higher basal level of hypodiploidy, as shown in control cells (Figure 4c, CTR). It can be observed that peaks of hypodiploidy were well detectable in all samples in the left region of the red area of the histograms (Figure 4a,b), indicating that they presumably refer to PI-stained DNA from cells at an advanced, but not precisely identifiable and computable, state of nuclear disruption. In particular, these peaks are more evident in PB2/NO-IL-2 samples. With emphasis, in addition, that PI staining does not allow differentiation between still-living and early-necrotic cells, the conclusions of this set of experiments were as follows: (i) the ability of TBT to induce high levels of cell death in HTLV-1-infected cells was clearly confirmed; (ii) the specific type of cell death induced by TBT, as well as an undoubtable difference in sensitivity to TBT of IL-2-dependent and IL-2-independent HTLV-1-infected cells, could not be inferred by flow cytometry analysis of PI-stained cells only; (iii) further experiments using different techniques were necessary to address the issue of cell death induced by TBT in HTLV-1-infected cells.

### 2.5. Nuclear Morphology Alteration Induced by TBT in HTLV-1-Infected Cells

**Figure 5:**
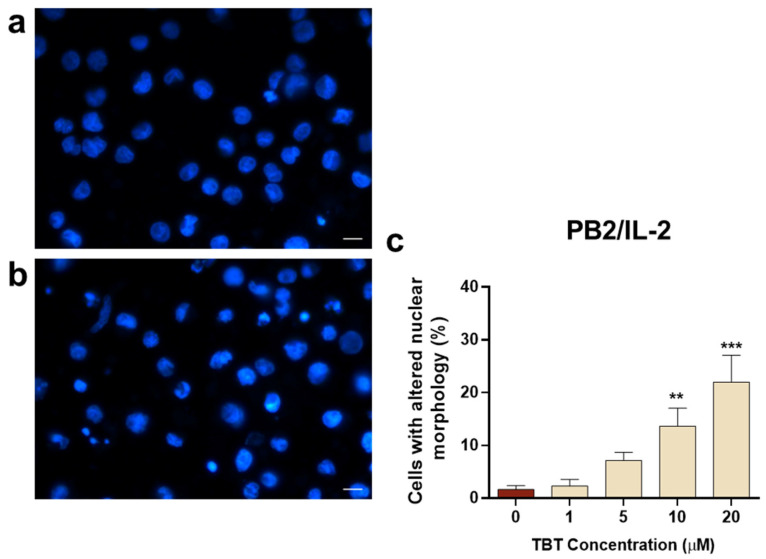
Nuclear morphology, as detected by the Hoechst stain technique, in HTLV-1-infected PB2/IL-2 cells at 6 h after treatment with TBT. The representative images show cells treated with control diluent (**a**) and cells treated with 10 μM TBT displaying typical features of nuclear alteration (**b**). The graph (**c**) represents the percentages of cells showing altered nuclear morphology after treatment with control diluent (0), 1, 5, 10, or 20 µM TBT. Data are expressed as mean values ± S.D. from three biological replicates. Asterisks indicate statistically significant comparisons versus control (** *p* ≤ 0.01; *** *p* ≤ 0.001). Images were acquired at 400× magnification; scale bar, 10 μm.

To further investigate cell death induced by TBT in HTLV-1-infected cells, the morphological changes caused by TBT were analyzed by fluorescence microscopy using the Hoechst staining technique. Based on results for cytotoxicity obtained in the WST-1 and Trypan Blue assays at 24 h post treatment, and for hypodiploidy obtained at 13 h post treatment, in this set of experiments, a shorter time course of 6 h was adopted. The representative images of Figure 5 show the appearance of PB2/IL-2 cells with altered nuclear morphology, including both chromatin condensation and nuclear fragmentation with formation of nuclear bodies following treatment with TBT 5 µM (Figure 5b), in comparison with control PB2/IL-2 cells (Figure 5a). A notable, dose-dependent increase in cells showing altered nuclear morphology following TBT treatment can be clearly observed in the numerical results represented in the graph (Figure 5c). Data obtained from three independent experiments show a significant and a highly significant increase in PB2/IL-2 cells with altered nuclear morphology following treatment with 10 µM TBT and 20 µM TBT, respectively, in comparison with control cells.

**Figure 6:**
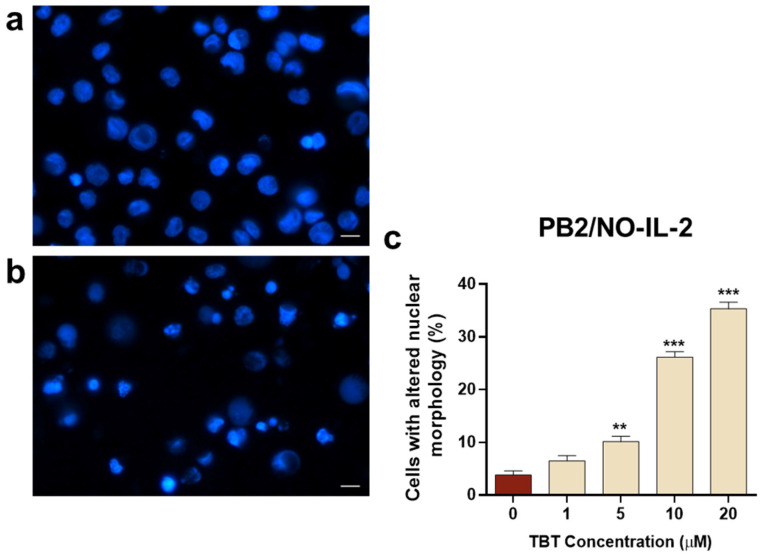
Nuclear morphology, as detected by the Hoechst stain technique, in HTLV-1-infected PB2/NO-IL-2 cells at 6 h after treatment with TBT. The representative images show cells treated with control diluent (**a**) and cells treated with 10 μM TBT displaying typical features of nuclear alteration (**b**). The graph (**c**) represents the percentages of cells showing altered nuclear morphology after treatment with control diluent (0), 1, 5, 10, or 20 µM TBT. Data are expressed as mean values ± S.D. from three biological replicates. Asterisks indicate statistically significant comparisons versus control (** *p* ≤ 0.01; *** *p* ≤ 0.001). Images were acquired at 400× magnification; scale bar, 10 μm.

**Figure 7:**
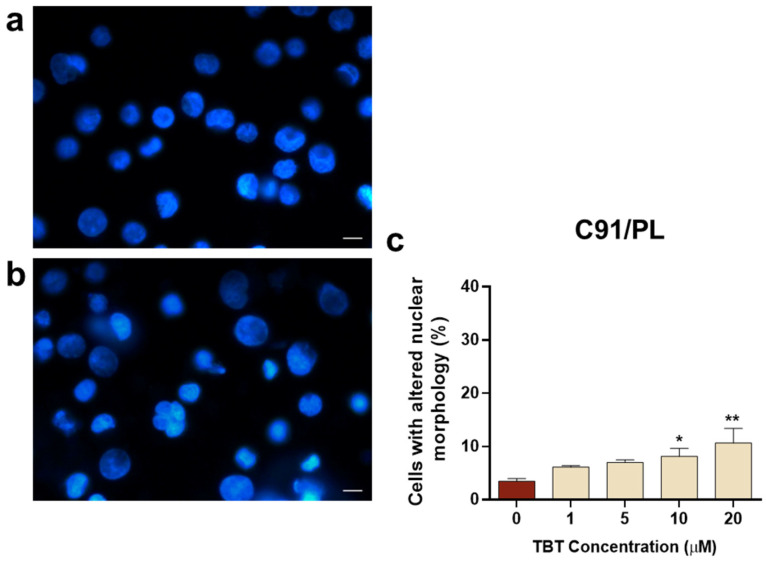
Nuclear morphology, as detected by the Hoechst stain technique, in the HTLV-1-transformed C91/PL cell line at 6 h after treatment with TBT. The representative images show cells treated with control diluent (**a**) and cells treated with 10 μM TBT displaying typical features of nuclear alteration (**b**). The graph (**c**) represents the percentages of cells showing altered nuclear morphology after treatment with control diluent (0), 1, 5, 10, or 20 µM TBT. Data are expressed as mean values ± S.D. from three biological replicates. Asterisks indicate statistically significant comparisons versus control (* *p* ≤ 0.05; ** *p* ≤ 0.01). Images were acquired at 400× magnification; scale bar, 10 μm.

**Figure 8:**
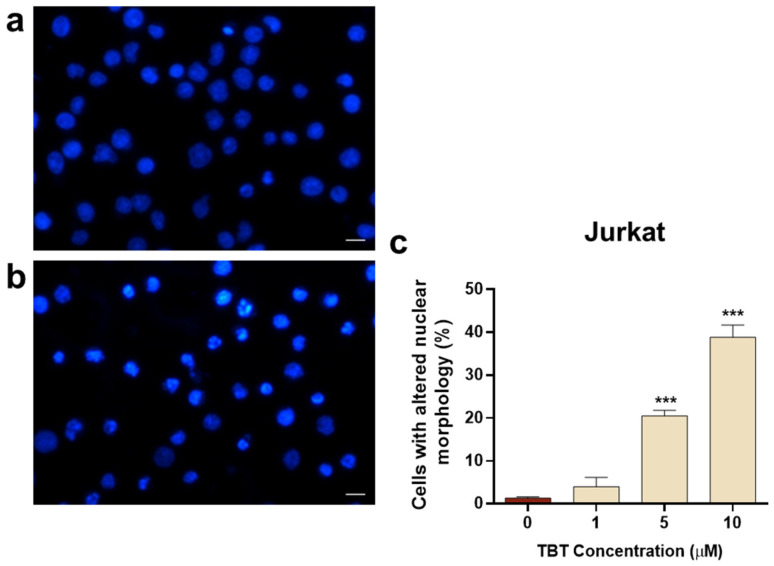
Nuclear morphology, as detected by the Hoechst stain technique, in the uninfected Jurkat cell line at 6 h after treatment with TBT. The representative images show cells treated with control diluent (**a**) and cells treated with 10 μM TBT displaying typical features of nuclear alteration (**b**). The graph (**c**) represents the percentages of cells showing altered nuclear morphology after treatment with control diluent (0), 1, 5, or 10 µM TBT. Data are expressed as mean values ± S.D. from three biological replicates. Asterisks indicate statistically significant comparisons versus control (*** *p* ≤ 0.001). Images were acquired at 400× magnification; scale bar, 10 μm.

The effects of TBT on nuclear morphology were also investigated in PB2/NO-IL-2 cells and in the HTLV-1-transformed C91/PL cell line. Regarding the PB2/NO-IL-2 cell line, results in Figure 6 show that, overall, these cells were more susceptible to the action exerted by the organotin molecule with respect to PB2/IL-2 cells, since they showed a nuclear morphology alteration in response to TBT, with a statistically significant difference in respect to control cells, already detectable at a concentration of 5 µM at the selected early time point. Moreover, the dose-dependent increase in cells with altered nuclear morphology at 10 µM TBT and 20 µM TBT was higher in comparison with PB2/IL-2 cells treated with the same concentrations of TBT (Figure 5c and Figure 6c). The susceptibility of C91/PL cells to TBT exhibited a different characteristic with respect to PB2/NO-IL-2 cells. Results shown in Figure 7 highlight how the organotin compound was unable to give rise to cells with morphologically altered nuclei at a concentration of 5 µM, as is shown in Figure 7b versus Figure 7a, and that a significant and a highly significant difference with respect to control cells were actually obtained in the cases of treatment with 10 µM and 20 µM TBT, respectively (Figure 7c). However, the percentage values of cells with altered nuclei were evidently lower than those obtained with the same concentrations of TBT in PB2/NO-IL-2 cells (Figure 6c). Uninfected Jurkat T cells subjected to the same experimental protocol adopted for HTLV-1-infected cells did not show altered nuclear morphology at 1 µM TBT, but a highly significant difference in comparison with control cells at 5 µM and 10 µM TBT was observed (Figure 8c). Note that in this case, 10 µM TBT was the highest concentration at which credible results could be reported because an elevated number of non-microscope-slide-adherent cells, presumably undergoing primary or secondary lytic cell death, was detected at a concentration of 20 µM TBT.

### 2.6. Effects of Caspase Inhibitors on Nuclear Morphology Alteration and Evaluation of Caspase-3 and Caspase-8 Activity Through Enymatic Assays in HTLV-1-Infected Cells Following Treatment with TBT

To obtain basic information on the characteristics of cell death caused by TBT in HTLV-1-infected cells at different stages of immortalization/transformation, first, the morphological changes caused by TBT in PB2/IL-2 and PB2/NO-IL-2 cells were analyzed again using the Hoechst stain technique, this time in cells subjected or not subjected to pre-treatment with caspase inhibitors— protease enzymes that play essential roles in various forms of regulated cell death (RCD), particularly apoptotic cell death. Cells were treated with 10 µM TBT alone or following a pre-treatment with the pan-caspase inhibitor (Z-VAD) or the caspase-3 inhibitor (Ac-DEVD). Pre-treatment with 25 µM Z-VAD was performed 1h before addition of TBT, with 0.1 µM Ac-DEVD, 15 min before the addition of the organotin compound, in accordance with routine methods.

**Figure 9:**
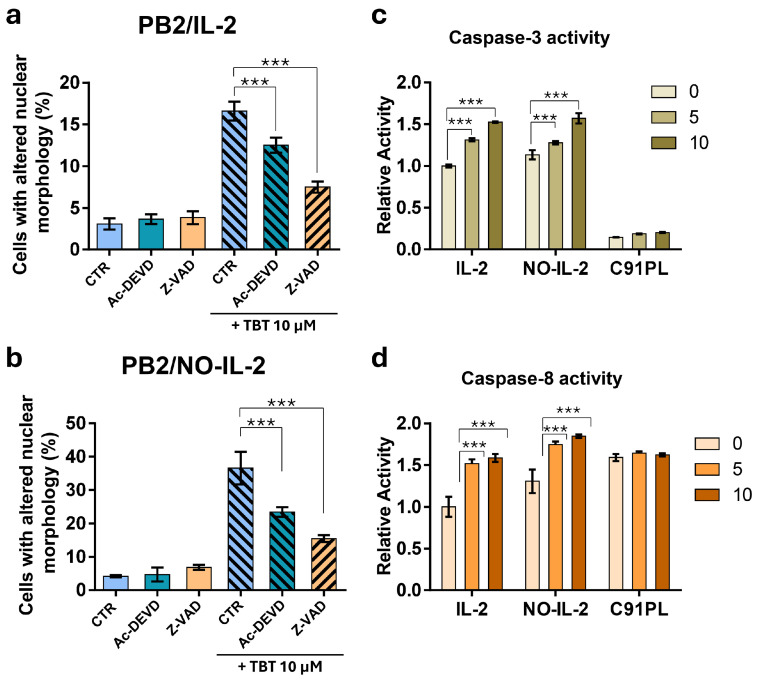
Effects of pre-treatment with caspase inhibitors on alteration of nuclear morphology induced by TBT and of treatment with TBT on caspase-3 and caspase-8 enzymatic activity in HTLV-1-infected cells. The percentages of PB2/IL-2 cells (**a**) or PB2/NO-IL-2 cells (**b**) showing altered nuclei morphology detected by the Hoechst stain technique after pre-treatment or no pre-treatment (CTR) with Z-VAD or Ac-DEVD and then treated with 10 µM TBT are reported. Relative caspase-3 (**c**) and caspase-8 (**d**) enzymatic activity detected by colorimetric assay kits in PB2/IL-2 (IL-2) and PB2/NO-IL-2 (NO-IL-2), as well as in HTLV-1-transformed C91/PL cells, after treatment for 2 h with 5 and 10 µM TBT, are reported. The data in the graph are expressed as mean ± S.D. obtained from three biological replicates. Asterisks at the top of the bars indicate statistically significant comparisons between marked groups (*** *p* ≤ 0.001).

The results shown in Figure 9a reveal a notable, highly significant reduction in PB2/IL-2 cells with altered nuclear morphology following pre-treatment with the pan-caspase inhibitor, in comparison with cells treated with TBT alone. This phenomenon can be clearly observed in the numerical results represented in the graph. Regarding PB2/IL-2 cells pre-treated with Ac-DEVD, results showed a partial, apparently lower than that observed with Z-VAD, reduction in cells with altered nuclear morphologies. Nevertheless, also in this case, the difference versus control cells was highly significant. When PB2/NO-IL-2 cells were subjected to the same protocol of pre-treatment with caspase inhibitors utilized for PB2/IL-2, the results showed that Z-VAD was able to cause a clear, highly significant reduction in cells showing nuclear morphology alteration following TBT treatment (Figure 9b). As concerns pre-treatment with Ac-DEVD, the results reported in the graph show an evident, highly significant reduction in cells with altered nuclear morphologies for samples treated with TBT (Figure 9b). However, also in this case, the relative reduction was lower than that observed with Z-VAD, indicating that the effect of the pan-caspase inhibitor could not be attributable merely to the inhibition of caspase-3. In all experiments performed using caspase inhibitors, both in PB2/IL-2 and PB2/NO-IL-2 cells, the caspase inhibitors alone did not modify the nuclear morphology.

To further investigate the possible mechanisms underlying the RCD induced by TBT, direct evaluation of caspase-3 and caspase-8 activation was performed in HTLV-1-infected cells following treatment with the organotin compound. A total of 2 × 10^6^ cells/sample of PB2/IL-2, PB2/NO-IL-2, and C91/PL cells were treated with 5 μM and 10 μM TBT, respectively, for 2 h. Cell lysates were incubated with the substrate Ac-IETD-pNA, and the generated yellow pNA (p-Nitroaniline) was measured at 405 nm by a spectrophotometer. The same reaction was set for the diluent-treated control cells, the treated samples, and for the blank. The activation of caspase activity was evaluated arbitrarily assuming that in the PB2/IL-2 diluent-treated samples there was a value of 1 for caspase-3 and caspase-8 activation. As shown in Figure 9c, in the PB2/IL-2 cell line, after 2h of treatment with 5 μM and 10 μM TBT, a highly significant (*** *p* ≤ 0.001) increase in the activated form of caspase-3 with respect to control cells was detected. Similarly to caspase-3, even caspase-8 was activated following treatments with 5 μM and 10 μM TBT in a dose-dependent fashion in PB2/IL-2 cells (Figure 9d). In PB2/NO-IL-2, a similar trend of a highly significant increase in the activated forms of both caspase-3 and caspase-8 following TBT treatment was detected with respect to PB2/IL-2 cells, with an apparently more pronounced effect for caspase-8 activation (Figure 9c,d). In the C91/PL-transformed cell line, which showed resistance to cytotoxicity induced by TBT, no sign of either caspase-3 or caspase-8 activation in respect to control cells was detected after treatment with TBT (Figure 9c,d). Note that in the latter cell line, showing very low levels of cell death following TBT treatment, basal levels of caspase-3 activation were very low, while, in contrast, basal levels of caspase-8 activation, always with reference to the value of 1 in PB2/IL-2 cells, were very high.

### 2.7. Time-Course Analysis of Cell-Death-Related Events with a Live-Cell System via Real-Time Quantification of Fluorescent C91/PL Cells Double-Stained with Green Caspase-3/7 and Red Cytotox Dyes

**Figure 10:**
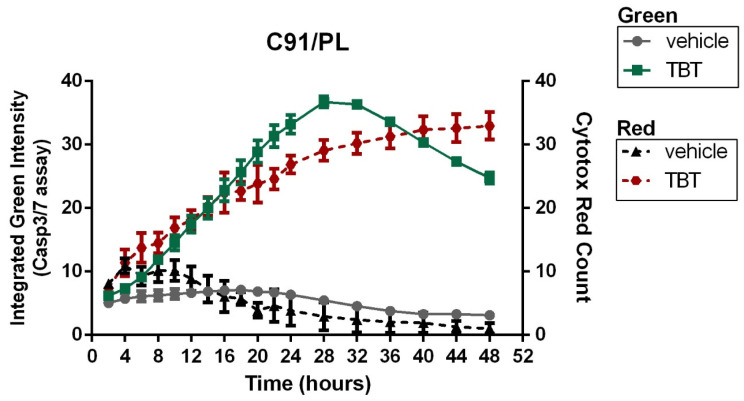
Real-time kinetic analysis of cell death in C91/PL cells treated with TBT using a live-cell imaging system. C91/PL cells were seeded at 5 × 10^4^ cells/well and treated with TBT (10 µM) or vehicle control, then double-stained with CellEvent™ Caspase-3/7 Green Detection Reagent and Incucyte^®^ Cytotox Red Dye and monitored in real time using the IncuCyte^®^ SX1 live-cell analysis system. Green fluorescence (solid lines) reflects Caspase-3/7 activation associated with apoptotic cell death, while red fluorescence (dashed lines) reflects loss of plasma membrane integrity associated with lytic cell death. Fluorescent signals were quantified using the integrated IncuCyte^®^ analysis software and normalized to cell count to account for changes in cell number over time. For graphical representation, green fluorescence values were divided by 100,000 and red fluorescence values by 10,000 to allow simultaneous visualization on dual y-axes. Data are shown as mean ± S.D. from three biological replicates.

Considering the difficulties encountered in detecting cell-death-related events at the same time utilizing different assays, each of which having different optimal times, and that the results obtained indicated how cytotoxicity exerted by TBT presumably involved different types of cell death, each of which with different kinetics of occurrence, we then decided to perform a more informative and advanced experimental activity. For this purpose, C91/PL cells, treated or not treated with TBT at the cytotoxic concentration of 10 µM, were subjected to a real-time evaluation of apoptotic and lytic cell death parameters, respectively, following double staining with green fluorescent Caspase-3/7 dye and red fluorescent Cytotox dye, as well as live-cell monitoring in the culture of fluorescent cells. The results, illustrated in the graph in Figure 10, show overall stable or slightly decreasing green and red fluorescence in vehicle-treated cells over the entire observation period, indicating negligible or absent levels of spontaneous cell death in C91/PL cells during the first 48 h in culture. Conversely, the curve of TBT-treated C91/PL cells shows an early and remarkable increase in green fluorescent cells, with a peak at about 24/28 h after treatment before a subsequent progressive decrease in green fluorescent cells. This kinetic clearly matches with the classic kinetic of apoptotic cell death and successive secondary necrosis occurrence. In parallel, the curve of red fluorescent cells undergoing lytic cell death also shows an increasing trend, even if slighter than that observed for green fluorescent cells, from the beginning of the observational time in culture. However, in this case, no peak or decreasing trends were observed at 24/28 h after treatment, instead a continuous rise in the curve throughout the period of observation in culture. This is consistent with the occurrence of a lytic type of cell death from the beginning of the administration of TBT in the cell culture, presumably enforced by occurrence of secondary necrosis starting from 24 h after treatment in cells that had undergone apoptotic RCD.

## 3. Discussion

There are currently no therapies or vaccines capable of controlling or slowing the progression of syndromes associated with HTLV-1 infections, although HTLV-1 continues to spread in many parts of the world and numerous cases of correlated rare, but very severe, diseases continue to be found. Hence, there is a constant need to find new therapies. Until now, many studies have demonstrated the high toxicity of organotin compounds toward tumor cells, with differences depending on the functional groups linked to the metal [33]. No data, however, are available on the effect of organotin compounds on HTLV-1-infected cells. Thus, obtaining preliminary information on the effects of an organotin-based compound on HTLV-1-infected cells has been chosen by us as the main subject of this study. Notably, also based on recent findings that various molecules containing the tin metal in their structure could exhibit potential anti-cancer activity causing a caspase-3 dependent cell death [34], our study aimed to investigate the in vitro activity toward HTLV-1-infected cells of a synthetic compound containing the tin molecule linked to three butyls and a trifluoroacetate chosen as the prototype for this study.

After years of failure in finding therapeutically active tools able to cure advanced ATL, and considering the increasing interest in a possible early intervention before diseases caused by HTLV-1 become much too aggressive [35], an original feature of our study was the preliminary setup and characterization of two in-house-generated cell lines from the same healthy donor mimicking two different early stages of HTLV-1-driven leukemogenesis. These cell lines, together with the C91/PL cell line, were then utilized as targets for evaluating the cytotoxic potential of the chosen tin-derivative prototype compound. Interestingly, the preliminary phenotypic characterization of the two generated HTLV-1-infected cell lines gave reasonable evidence of a more advanced process of immortalization/transformation for PB2/NO-IL-2 cells with respect to PB2/IL-2 cells. A higher expression level of markers, such as CD69, HLA-DR, and CD25, related to T-cell activation was found in PB2/NO-IL-2 and, intriguingly, this rendered the IL-2-independent cell line more similar to the C91/PL-transformed cell line than the PB2/IL-2 cell line from a phenotypical point of view. Thus, the preliminary research activities of the present study presented the possibility of having three different cell lines infected by HTLV-1 but presumably corresponding to different stages of the immortalization/transformation process as experimental tools. This possibility allowed us to mimic in some way the situation occurring in vivo in some patients with HTLV-1 infection in which their T cells may gradually undergo the different phases of the long pre-malignant process leading to ATL, and to test whether the cells at different stages of transformation might respond differently to treatment with the organotin compound. Similar experimental models are not usually used to identify new molecules possibly acting as pharmacological agents to contrast leukemogenesis caused by HTLV-1 (they are complex, long, and troublesome to set up). Regarding the phenotypic characterization of the three models of HTLV-1-infected cell lines utilized and relevant to the main aim of this study, finally, it is interesting to note that the fully transformed C91/PL cell line showing the highest T-cell activation marker expression also exhibited the highest level of resistance to TBT-induced cell death compared to the pre-malignant PB2 cell lines. This is consistent with the well-known scenario in which the cellular transcriptional activation triggered by viral oncogenic genes plays a crucial role in the mechanisms underlying leukemogenesis induced by HTLV-1 infection of T cells [36].

Regarding the tin-derivative compound, a previous study by us demonstrated that TBT, a non-commercially available, prototype synthetic compound, was endowed with a cell toxicity higher than that of cisplatin toward various tumor cell targets, and that its toxicity was not related to the interaction with DNA, but presumably to the inhibition of glucose uptake into the cells [32]. The exact mechanisms underlying this phenomenon, however, were not ascertained. When, in this study, the HTLV-1-immortalized/transformed cell lines and a lymphoid, uninfected cell line were subjected to TBT treatment followed by an accurate investigation of cytotoxicity induced using two classic techniques, such as the detection of metabolic activity and of live/dead cells by the Trypan Blue exclusion test, the results highlighted a potent toxic effect at concentrations equal to or greater than 10 µM at a prolonged time of 24 h. Notably, the latter test showed that the number of dead cells never mirrored that of live cells, indicating that quantitative results of this assay should not precisely reflect the real toxicity of TBT. The results, indeed, also showed that uninfected cells, including peripheral blood mononuclear cells from a healthy donor, suffer from cytotoxicity induced by TBT. Nevertheless, analysis of IC50 values indicated that uninfected cells were not more sensitive, but rather slightly more resistant to TBT than infected cells. In fact, the Selectivity Index (SI), calculated as the ratio of IC50 values detected by the WST-1 assay in uninfected cells to those detected in HTLV-1-infected cells, was always higher than 2 for all three experimental models of HTLV-1-infected cell lines assayed. And, in particular, PB2/NO-IL-2 showed an SI of 3.9 and 4.3 in comparison to uninfected Jurkat T cells or PBMCs from a healthy donor, respectively, proving to be the most resistant to TBT of all the cells assayed. In this regard, we must actually point out that only three non-zero point concentrations are not the optimum to calculate exact IC50 and related SI values. Therefore, this is a limitation of our study. In fact, the high and immediate cytotoxicity exerted by TBT on the four cell lines subjected to the WST assay (close to zero at 1 μM for the more resistant cell lines, and close to 100% at 10 μM for the more sensitive cell lines), greatly restricted the range of concentrations useful for comparing their SI. Nevertheless, given that the IC50 values for all the cell lines utilized for the comparison were well within the range of the concentrations utilized for their calculations, this suggests considering them, even if not strictly exact, very representative of the different sensitivity of the cell lines to TBT cytotoxicity. In any case, the cell destruction caused by TBT at these doses and times of treatment toward both infected and uninfected cells was somewhat massive, indicating the lack of an adequate therapeutic window for this molecule. This initial screening, then, suggests the need for profound chemical refinements to the molecular structure of TBT principally aimed at reducing cytotoxicity on uninfected cells without losing activity toward HTLV-1-infected cells. Such a future action is necessary to generate TBT-derived molecules provided in a reasonable therapeutic window useful to contrast leukemogenesis caused by HTLV-1. This caused us to concentrate our efforts, at this stage, on better analyzing the possible mechanisms involved in the cytotoxic effects of TBT and on shorter and lower-dose treatments in the successive experimental phases.

Based on previous experience with tumor cell lines, a first approach consisted of using flow cytometry analysis following PI staining to detect apoptotic-type cell death. Unfortunately, however, although our results indicated that apoptosis was presumably involved in the dramatic cytotoxic effects induced by TBT, flow cytometry analysis of hypodiploidy outlined a complex and presumably not unique overall picture concerning cell death induced by TBT in HTLV-1-infected cells. Yet, these experiments suggested that a difference, though not easily quantifiable, could exist between IL-2-dependent and IL-independent cell lines, both in basal levels and in TBT-induced levels of cell death. This prompted us to carry on further experiments to address the cell death characterization.

When, in even more pronounced short-term conditions, the Hoechst staining technique followed by fluorescence microscopy analysis was utilized, the results confirmed the ability of TBT to exert a cytotoxic effect on HTLV-1-infected and uninfected cells, showing induction of cell death characterized by evident nuclear morphology alterations. Notably, it is well known that the Hoechst staining technique, different to flow cytometry analysis of PI-stained cells, allows for visual exclusion of debris and discrimination of cells that underwent apoptotic cell death from living cells and from cells that underwent necrosis by means of the detection of nuclei usually showing condensation and fragmentation [37]. In fact, such features were detectable in HTLV-1-infected and uninfected samples treated with TBT. Moreover, the Hoechst staining followed by fluorescence microscopy analysis allowed us to clearly confirm different, cell-dependent responses to treatment with TBT. IL-2-independent PB2/NO-IL-2 cells demonstrated a greater susceptibility to the cell-death-inducing effect of TBT with respect to the IL-2-dependent PB2/IL-2 cell line. This can possibly be deciphered as a protective effect toward TBT-induced cell death exerted by the presence of IL-2 as growth factor in the medium for PB2/IL-2 cells. Conversely, this difference could be attributed to a higher sensitivity of PB2/NO-IL-2 cells to TBT during their pre-malignant, evolutionary adaption in vitro to the lack of IL-2 as a growth factor, because of a not-yet-completed, HTLV-1-driven selection of cells all endowed with high resistance to cell death. Coherently with the above scenario, for what concerns the C91/PL cell line, the results in general showed a strong resistance to induction of cell death by the organotin compound.

Another aspect to point out concerning the results we obtained using either flow cytometry analysis of hypodiploidy or the Hoechst staining technique is that during the different phases of the process that within about 24 h leads to apoptosis, by means of both these techniques, a clear discrimination among living cells, apoptotic cells, or cells subjected to primary or secondary necrosis and simply debris could not be always performed. The different timings that we used for the two techniques were arbitrarily chosen based on preliminary results, but based on these experiments, we could not exclude that at even slightly different times, a slightly diversified situation in some of the experimental groups might be detected. Moreover, in recent years, the scenario of cell death has been enriched with several different forms of RCD that could also coexist in response to various stimuli [38,39], further complicating the exact identification of cell death type by a unique morphological assay such as the Hoechst staining technique [37]. This is the reason why, in the context of the experimental methodology and related results of the present study, we preferred not to simply use the terms “apoptosis/apoptotic cells”, but rather “hypodiploidy, nuclear morphology alteration, chromatin condensation-fragmentation, and cell death” to describe the cell-death-related effects caused by TBT in HTLV-1-infected or uninfected cells. In addition, this is also the reason why we decided to obtain more information on possible mechanisms involved in cell death caused by TBT using even more methodological approaches for better classifying cell death induced by TBT in HTLV-1-infected cells. In fact, experiments carried out with a pan-caspase inhibitor showed a clear involvement of caspase proteases on the mechanism involved in the action of the compound. At the same time, however, experiments using a specific caspase-3 inhibitor showed only a partial inhibition of nuclear morphology alteration induced by co-treatment with TBT with respect to that induced by the compound alone, suggesting that it is likely that not only caspase-3 is involved in cell-death-related effects induced by the compound. Moreover, to further investigate the characteristics of cell death induced by TBT, we also performed additional experiments in C91/PL HTLV-1-transformed cells using a more functional, quantitatively reliable, and temporally relevant technique, i.e., the real-time simultaneous monitoring of apoptotic-like and lytic-like cell death via automatic evaluation of green and red fluorescence of cells in culture following staining with Caspase-3/7 dye and Cytotox dye, respectively. Thanks to this experimental approach, we confirmed substantial results obtained via the other techniques indicating the presence of different types of cell death in HTLV-1-infected cells following TBT treatment, also reinforcing them with a clear kinetic picture of the observed phenomena. These types of cell death include, on the one hand, Caspase-3/7-dependent cell death, and on the other hand, occurrence of both early and late lytic cell death.

## 4. Materials and Methods

### 4.1. Tributyltin Trifluoroacetate and Chemicals

Tributyltin trifluoroacetate, termed TBT in this study, is an organic compound containing tin synthesized at the Department of Chemistry “Ugo Schiff” of the University of Florence as previously described [32]. TBT was chosen as the prototype compound for this study considering its adequate chemical properties for in vitro cellular treatment described in detail in the previous study [32].

General formula: Bu_3_SnOCOCF_3_

Molecular formula: C_14_H_27_F_3_O_2_Sn

Molecular weight: 403.07 g/mol

TBT was diluted in DMSO (cat. S-D2650, Sigma, Saint Louis, MO, USA) up to a final concentration of 1M and stored at −20 °C before its utilization for the biological assays. In all experiments carried out with TBT, the control cells were exposed for the same amount of time as the treated cells to control diluent alone, corresponding to the higher concentration of the compound assayed.

Z-VAD-FMK (cat. tlrl-vad, Invivogen, San Diego, CA, USA) is a cell-permeant irreversible pan-caspase inhibitor. It was diluted in DMSO up to a final concentration of 20 mM and stored at −20 °C. The final concentration used for experiments was 25 μM, added after a pre-treatment of 1 h.

Ac-DEVD-CMK (cat. 218750, Millipore, Burlington, MA, USA) is a cell-permeable and irreversible inhibitor of the cysteine protease caspase-3. It was diluted in DMSO up to a final concentration of 100 mM and stored at −20 °C. It was tested at 0.1 μM concentrations and was added 15 min before the organotin treatment.

### 4.2. Cells

The PB2/IL-2 cell line was generated in our laboratory by co-culturing peripheral blood mononuclear cells (PBMCs) isolated from a single healthy adult donor with HTLV-1-chronically infected MT-2 cells. MT-2 cells were irradiated with 120 Cy immediately before the co-culture with PBMCs at a ratio of 5:1, to allow HTLV-1 cell-to-cell transmission but also extinction of the MT-2 donor cells a few weeks later [40]. The co-culture was set up in complete medium (CM) consisting of RPMI (Corning, Tewksbury, MA, USA) culture medium with L-glutamine, 25 mM Hepes, 50 U/mL penicillin, and 50 U/mL streptomycin (Corning), with the addition of 10% fetal bovine serum FBS (Corning). The growth factor IL-2 (Proleukin, Chiron, Amsterdam, The Netherlands) was added to the co-culture and was first examined about four weeks after the setup. An initial check on HTLV-1 infection and cell growth was carried out, and the presence of immortalized or dead cells due to a process of cell necrosis or apoptosis was evaluated [41]. PB2/IL-2 HTLV-1-infected, immortalized cells were maintained in continuous culture in the presence of IL-2 for 12 months before they were amplified, divided into different samples, and frozen until their usage. The PB2/NO-IL-2 cell line was generated in our laboratory from PB2/IL-2 cells. The independence from the growth factor was gradually obtained in some PB2/IL-2 cultures grown at a density of 5 × 10^5^ cells/mL via the progressive addition of decreasing, lower units of IL-2 until addition of IL-2 was completely omitted and a complete IL-2 independence was achieved. Cells that survived this selection procedure were then maintained in continuous culture in the absence of IL-2 and stabilized for a further 12 months before they were amplified, divided into different samples, and frozen until their usage. For carrying on the experimental activities, PB2/IL-2 and PB2/NO-IL-2 cells were thawed and grown in suspension culture at a density of 4.5 × 10^5^ cells/mL in the presence or the absence of 20 units of interleukin-2/mL, respectively.

The HTLV-1-transformed C91/PL cell line was stabilized toward the end of the 1980s in Japan, starting from a co-culture of umbilical cord cells with lymphomonocytes of a patient with HLTV-1-positive lymphoma. The C91/PL cell line was grown at a density of 4 × 10^5^ cells/mL.

The lymphoid uninfected Jurkat T-cell line was grown in suspension in CM at a density of 2.5 × 10^5^ cells/mL.

All the cell lines used for this study were grown at 37 °C in a humidified atmosphere with 5% CO_2_ with or without the addition of TBT at different concentrations.

The PBMCs utilized in some experiments were isolated from the buffy coat collected from healthy adult donor volunteers, who were seronegative for HIV and hepatitis B and C viruses, enrolled in the Polyclinic Hospital Tor Vergata Transfusion Center for blood donation for therapeutic purposes. The donors authorized the use of the remaining leukocytes for research purposes, signing a consent form. Anonymized buffy coats were diluted in phosphate-buffered saline at pH 7 (PBS), and mononuclear cells were separated using a Ficoll–Hypaque density gradient (Cederlane, Hornby, ON, Canada) at a cells/gradient ratio of 1:2. The cells were then centrifuged for 30 min at 1800 RPM and washed twice in RPMI 1640 medium (Corning). The PBMCs were then subjected to phenotypic characterization or stimulated with IL-2 at 10 U/mL (Proleukin) before treatment with the compound under study.

### 4.3. Metabolic Activity and Viability Assays

The inhibition of cell metabolic activity induced by the tested compound was evaluated using the WST-1 colorimetric assay. This method relies on the reduction of the stable tetrazolium salt WST-1 into a soluble formazan dye through a complex cellular mechanism that mainly takes place at the cell surface. The bioreduction process depends on the generation of NAD(P)H by viable cells during glycolysis. Consequently, the amount of formazan formed is directly proportional to the number of metabolically active cells that are also potentially capable of proliferation. Quantification of the produced formazan is achieved by measuring the absorbance of the dye solution at 450 nm using a multi-well spectrophotometer (SpectraCount, Packard Instrument Company Inc., Meriden, CT, USA). For the assay, cells were seeded at a density of 6 × 10^4^ per sample in each well of a flat-bottom 96-well plate (Corning) with a final volume of 100 μL. Treatments were then applied, and the plate was incubated at 37 °C. After a 24 h incubation period to assess cell viability, 10 μL of WST-1 reagent (cat. CELLPRO-RO, Roche Diagnostics GmbH, Mannheim, Germany) was added to each well, followed by a further incubation period to allow color development, indicating metabolic activity. Finally, absorbance values were recorded at 450 nm using a microplate reader, with background controls included for reference.

The viability assay was performed using the Trypan Blue (cat. T8154, Sigma) exclusion test after a 24 h incubation period, with the concentrations of the compound under study selected based on the WST-1 assay and preliminary Trypan Blue exclusion tests.

### 4.4. Phenotypic Characterization by Immunostaining and Flow Cytometry Analysis

For the analysis of the basic T-cell phenotype and the T-cell clusters of differentiation markers, approximately 1 × 10^6^ cells were suspended in 100 µL of PBS and incubated at 4 °C for 30 min with the following antibodies: anti-human CD3 BV785 (cat. 317330, Biolegend, San Diego, CA, USA); anti-human CD4 PerCP/cy5.5 (cat. 317428, Biolegend); anti-human CD25 APC (cat. 284549, Sony Biotechnology, Gauteng, South Africa); anti-human CD69 BV605 (cat. 310938, Biolegend); anti-human CD8 APC750 (cat. 344746, Biolegend); anti-human CD43 PE/Cy7 (cat. 343208, Biolegend); anti-human CD95 FITC (cat. 555673, BD Pharmingen, San Diego, CA, USA); anti-human HLADR PE (cat. 32661, BD Bioscences, San Jose, CA, USA). All the stained cells were analyzed via CytoFLEX (Beckman Coulter) and the CytExpert 2.0 software (Beckman Coulter).

### 4.5. Evaluation of Cell-Death-Related Events

Early cell-death-related hypodiploidy, usually utilized as an apoptotic-RCD biomarker, was assessed by flow cytometry analysis using a CytoFLEX (Beckman Coulter, Brea, CA, USA) on ethanol-fixed and -permeabilized cells stained with propidium iodide (PI) (Merck KGaA, Darmstadt, Germany). Data acquisition and analyses were performed using CytExpert 2.0 (Beckman Coulter) on a minimum of 150,000 events for each sample.

Nuclear morphology alteration, mainly consisting of chromatin condensation and nuclear fragmentation with formation of nuclear bodies assumed as typical apoptotic-RCD features, was evaluated via morphological analysis of cells following staining with Hoechst chromatin dye and microscopy analysis, as previously described [42].

### 4.6. Evaluation of Caspase-3 and Caspase-8 Activity

The activation of caspase proteases 3 and 8 was evaluated using colorimetric assay kits (cat. E-CK-A383, cat. E-CK-A388, Elabscience, Houston, TX, USA), following the manufacturer’s instructions. The assay is based on the cleavage of a caspase-sequence-specific substrate conjugated to the chromophore p-nitroaniline (pNA). Upon activation, the caspase enzyme cleaves the substrate, releasing the yellow-colored pNA, whose absorbance can be measured spectrophotometrically.

For each condition, lysates were prepared from 2 × 10^6^ cells per sample after 2 h of treatment with the TBT compound at different concentrations. Following incubation with the caspase-specific pNA-conjugated substrate for 2/4 h, the absorbance of the released pNA was measured at λ = 405 nm using a spectrophotometer. The obtained optical density (OD) values were normalized to total protein content, as determined by the Bradford assay (cat. 5000001, Bio-Rad. Hercules, CA, USA), and expressed as percentage values relative to control samples.

### 4.7. Real-Time Analysis of Cell Death Using IncuCyte^®^ Live-Cell Imaging

Real-time monitoring of cell-death-related events was performed using the IncuCyte^®^ SX1 live-cell analysis system (Sartorius, Ann Arbor, MI, USA) equipped with the IncuCyte^®^ software version 2023A. C91/PL cells were seeded in 96-well plates at a density of 5 × 10^4^ cells per well in RPMI with 5% FBS and allowed to equilibrate before treatment. Cells were treated with TBT at a final concentration of 10 µM or with vehicle control (DMSO at the corresponding final concentration). To simultaneously monitor regulated and lytic cell death, cells were double-stained with CellEvent™ Caspase-3/7 Green Detection Reagent (Invitrogen™, Thermo Fisher Scientific, Waltham, MA, USA, Cat. No. C10427) and IncuCyte^®^ Cytotox Red Dye (Sartorius, Cat. No. 4632), added directly to the culture medium according to the manufacturers’ instructions. The CellEvent™ Caspase-3/7 reagent is a cell-permeant fluorogenic substrate containing the DEVD peptide sequence that, upon cleavage by activated caspase-3 and -7, binds DNA and emits green fluorescence (Ex/Em ~511/533 nm), thereby selectively labeling apoptotic cells. The IncuCyte^®^ Cytotox Red Dye is a membrane-impermeant nucleic acid dye that enters cells only upon loss of plasma membrane integrity, producing a strong red fluorescent signal indicative of lytic cell death. Plates were placed in the IncuCyte^®^ instrument housed within a standard humidified incubator at 37 °C and 5% CO_2_. Phase-contrast and fluorescence images were acquired using a 10× objective, collecting three non-overlapping fields per well. Image acquisition was performed every 2 h during the first 24 h and every 4 h thereafter up to 48 h. Quantification of green and red fluorescent objects was performed using the integrated IncuCyte^®^ analysis software. For combined graphical representation of green and red fluorescence kinetics, green fluorescence values were scaled by division by 100,000 and red fluorescence values by division by 10,000. Data are expressed as mean ± S.D. of three independent experiments.

### 4.8. Statistical Analysis

GraphPad Prism (version 9.0) software was used both for statistical analysis and for generating data plots. All experiments were performed in at least three independent biological replicates (*n* = 3), each analyzed in technical triplicates utilized as mean values, unless otherwise specified. Data are expressed as mean ± S.D. IC50 and CC50 values were calculated via nonlinear regression using a sigmoidal dose–response (log[inhibitor] vs. normalized response) model in GraphPad Prism. Data were fitted to a four-parameter logistic equation (variable response as a function of log concentration), and IC50/CC50 values were interpolated from the fitted curves. For Figure 2 and Figure 3, 95% confidence intervals (95% CI) were additionally computed and displayed to provide a numerical estimate of the precision of IC50/CC50 values. Differences across experimental conditions were analyzed with Student’s *t*-test or one-way ANOVA, as appropriate. When multiple comparisons were performed, one-way ANOVA was followed by Bonferroni-adjusted post hoc tests; comparisons were carried out between treated samples and the corresponding vehicle-treated controls, unless otherwise indicated in the figure legends. Statistical tests and significance thresholds used are reported in the corresponding figure legends where appropriate.

## 5. Conclusions

In conclusion, the impact of the ligand structure and mechanisms involved in the toxicity of organotin compounds remain to be clarified, but nevertheless, TBT might be the prototype of a family of agents whose capacity to contrast leukemogenesis driven by HTLV-1 infection must be proven. Overall, the results obtained during this study indicate that the tin-containing compound can induce in vitro high levels of a not-yet-well-characterized form of cell death, especially when used at low concentrations and short times of treatment in cells mimicking a pre-malignant phase of HTLV-1 infection. Actually, most of the cells in our IL-2-independent HTLV-1-infected cell line suffered from caspase-dependent RCD, showing altered nuclear morphology, but not all of them suffered from caspase-3-dependent, classic apoptotic RCD. Thus, even considering the results obtained with live-cell imaging in C91/PL cells, presumably, various types of unregulated or regulated forms of cell death contribute to the cytotoxic effects exerted by TBT. Further studies, already underway in our laboratories, are necessary to better characterize cell death induced by organotin compounds in HTLV-1-infected cells. However, we are aware that the results reported in this study underscore the long process necessary to potentially transform TBT into a novel potential lead compound whose actual ability to contrast HTLV-1-induced leukemogenesis will need to be proven by following the experimental preclinical pathway necessary to deduce its effects and effectiveness in relevant in vivo models. Nevertheless, we hope that the novel pharmacological findings of this study can be translated into new therapeutic approaches to leukemia caused by HTLV-1 infection, thus contributing to the wellbeing of patients with HTLV-1 infection.

## Data Availability

Data are contained within the article.

## Abbreviations

The following abbreviations are used in this manuscript:

HTLV-1	T Human T-Lymphotropic virus type 1
TBT	tributyltin 2,2,2-trifluoroacetate
RCD	Regulated cell death
PI	Propidium iodide
